# Association of allostatic load measured by allostatic load index on physical performance and psychological responses during arduous military training

**DOI:** 10.14814/phy2.70273

**Published:** 2025-03-20

**Authors:** Evan D. Feigel, Kristen J. Koltun, Mita Lovalekar, Christopher K. Kargl, Matthew B. Bird, Jennifer N. Forse, Varun J. Patel, Brian J. Martin, Elizabeth F. Nagle, Karl E. Friedl, Bradley C. Nindl

**Affiliations:** ^1^ Neuromuscular Research Laboratory/Warrior Human Performance Research Center University of Pittsburgh Pittsburgh Pennsylvania USA; ^2^ Department of Health and Physical Activity University of Pittsburgh Pittsburgh Pennsylvania USA; ^3^ US Army Research Institute of Environmental Medicine Natick Massachusetts USA

**Keywords:** adaptation, Allostasis, fitness, maladaptation, stress

## Abstract

Military personnel experience decrements in physical fitness and psychological well‐being during training that may be attributed to allostatic load. This investigation examined the association between allostatic load measured by the allostatic load index (ALI) and physical performance and psychological responses in personnel undergoing a 10‐week training course. Thirty‐one participants (14 women) provided biochemical, questionnaire (perceived stress appraisal (PSS), sleep difficulty (SD), resilience (CD‐RISC‐25), and Physical Fitness Test (PFT; three‐mile run [3MR], pullups, Run‐Row PFT score, Push‐Pull PFT score, Crunches‐Plank PFT score, and total PFT score)) data before and after training. ALI (0–8) was calculated using biomarker components from neuroendocrine, autonomic, and immune systems. Simple linear regression analysis assessed the association between change (Δ) in ALI and responses. Backward stepwise regression identified components associated with responses (*α* = 0.05). In men, ΔALI was associated with Δpullups (*β* = −0.88, *p* = 0.015), Δpush‐pull PFT score (*β* = −2.87, *p* = 0.013), Δtotal PFT score (*β* = −3.48, *p* = 0.007), and ΔSD (*β* = −0.56, *p* = 0.046) with immune components explaining relationships. In women, ΔALI was associated with ΔSD (*β* = −1.25, *p* < 0.001) and ΔCD‐RISC‐25 (*β* = 2.65, *p* = 0.025) with no component explaining relationships. Increased ALI is associated with worsened physical performance in men and improved psychological outcomes in women, highlighting potential sex‐specific responses to increased allostatic load during training.

## 1 INTRODUCTION

Military training courses serve to prepare personnel for the physical and tactical requirements for successful military occupational role performance. Positive adaptation to the physical and psychological challenges involved in such courses, including energy deficiency (Alemany et al., 2008; Henning et al., 2014; Nindl et al., 1997), sleep deprivation (Gibson et al., 2022; Hansen et al., 2021), cognitive distress (Tait et al., 2024), and strenuous physical activity (Jurvelin et al., 2020; O'Leary et al., 2018), requires improvement in general and military‐specific physical fitness and psychological well‐being, including enhanced self‐appraised cognitive resiliency, reduced stress perception, and improvement in self‐reported sleeping difficulty level (Larsen et al., 2022). However, previous studies observed that personnel often experience maladaptive physical and psychological responses from training manifested in reductions in physical performance (Burley et al., 2018), self‐reported resiliency (Nindl et al., 2018), and sleep quality (Gibson et al., 2022), and increased stress perception (Bulmer et al., 2022). Such responses can increase the risk of illness (Billings, 2004), musculoskeletal injury (Orr et al., 2020), and attrition from the training course (Forse et al., 2024). Further, military personnel may be required to report to a subsequent duty station or deploy as soon as one week after graduation following course completion (Henning et al., 2014). This quick turnaround in occupational demands with limited time for appropriate recovery may necessitate adaptive responses during training. Understanding the biological processes associated with maladaptation may advance preventive means for enhanced post‐training occupational role performance.

Allostasis is a theoretical biological framework that outlines an adaptive regulatory process wherein physiological stability is maintained through the activation of stress‐related physiological systems that respond and adapt to physical, cognitive, and environmental stressors during predictable and unpredictable demanding life challenges (McEwen, 1998a, 1998b). Allostasis initiates from the activation of the hypothalamic‐pituitary‐adrenocortical (HPA) and the sympathetic‐adrenal‐medullary (SAM) systems that signal downstream physiological systems (i.e., immune and metabolic) to adapt to the challenge involved. This occurs from the activation of *primary mediators* characterized as biological factors from neuroendocrine, immunological, and autonomic systems that trigger downstream neurobehavioral and cardiometabolic effects to allow rapid physiological and behavioral adjustment to compromised physical, cognitive, or environmental demands. Deterioration of allostasis, however, may occur from repeated stress exposures or attempts at adaptation that require constant activation of mediators, which, when chronic, can result in allostatic load (McEwen, 1998a).

Allostatic load is a theoretical biological framework that outlines a maladaptive process wherein physiological stability fails owing to dysregulated mediator activity that results from malfunctioned feedback mechanisms and reflects the cumulative physiological burden in the face of chronic stress (McEwen, 1998b). Previous studies demonstrate that allostatic load can result in *secondary outcomes*, characterized as robust maladaptive physical and psychological outcomes reflected by worsened physical performance (Germano et al., 2023), sleep quality (Christensen et al., 2022), self‐appraised resilience (Felix et al., 2023), and perceived stress appraisal (McEwen, 2007). *Tertiary outcomes* can emerge from the consequences of secondary outcomes, such as pain syndromes, musculoskeletal injuries, and illnesses (Beckie et al., 2016; Feigel et al., 2024; Parker et al., 2022). Together, the allostatic load framework highlights the influence of the physical and social environment, individual variation in the stress response, and brain–body interactions to serve as a process by which chronic stress exposure may lead to robust maladaptation during military training (Feigel et al., 2024; McEwen, 1998b).

Allostatic load is quantified by its established traditional operationalization, the allostatic load index (ALI), which is a cumulative index score that reflects the extent of physiological dysregulation attributed to chronic stress exposure. The predominant approach to computing the ALI involves the calculation of individual mediator values into at‐risk quartiles associated with stress pathophysiology (i.e., upper or lower 25th percentile), scoring individuals with biomarkers lying within or beyond such quartiles, and summing across biomarkers to yield an ALI indicating the extent of allostatic load (Beckie et al., 2016). Biomarkers included in the ALI include mediators of HPA activity from the neuroendocrine system (i.e., cortisol and dehydroepiandrosterone), SAM activity from the autonomic nervous system (i.e., α‐amylase), and inflammatory activity from the immune system (i.e., interleukin‐6, interleukin‐10, C‐reactive protein, and tumor necrosis factor‐alpha) (McEwen, 1998b). Military personnel may be at risk for increased ALI when stress during training becomes additive or chronic, which may negatively impact post‐training occupational role performance.

Recent studies suggest that allostatic load serves as a useful model outlining a biological process driving maladaptive physical and psychological responses in personnel undergoing military training courses (Beckie et al., 2016; Bulmer et al., 2022; Feigel et al., 2024). However, whether the traditional measurement of allostatic load increases and is associated with robust measures of common maladaptive physical and psychological responses observed during training (i.e., *secondary outcomes*) remains uninvestigated. Additionally, previous studies report that the ALI delineates changes in physical and psychological function to a greater extent than singular biomarkers (Karlamangla et al., 2002). However, although the ALI demonstrates utility in predicting health outcomes in healthy and at‐risk populations (Juster et al., 2011; Karlamangla et al., 2002), it remains unclear whether this measure may appropriately represent the relevant contributions of primary mediators to military training‐related outcomes (Piazza et al., 2022). Indeed, previous studies observe that primary mediators of a system may be more responsive than others in relation to military‐specific training responses, such as elevated inflammatory cytokine concentrations in response to sleep disturbances and stress perception (Kargl et al., 2024), and blunted anabolic hormone concentrations in response to decrements in physical fitness (Booth et al., 2006; Tanskanen et al., 2011). Further, previous evidence suggests that mediator responses may be sex‐dependent (Conkright et al., 2022; Kargl et al., 2024). Hence, the individual or combinations of biomarker components of the ALI associated with physical and psychological outcomes by sex requires further investigation. Understanding the relationship between ALI and maladaptive physical and psychological responses, as well as examining those pertinent ALI components driving such relationships, may reinforce the allostatic load model as an underlying biological process associated with military training‐related maladaptation, elucidate by‐system specific ALI components driving such maladaptive outcomes, and support the development of preventative means to combat increases in allostatic load for enhanced post‐training occupational role performance.

The purpose of this investigation was to assess whether allostatic load, quantified by ALI, increased during the US Marine Corps Officer Candidate School (OCS) and determine the association between ALI and robust measures of physical performance and psychological responses during training. Additionally, this investigation aimed to elucidate those singular or combinations of biomarker components of the ALI associated with physical and psychological outcomes by sex. First, it was hypothesized that ALI would significantly increase upon completion of OCS in both sexes. Second, it was hypothesized that increased ALI would be positively associated with perceived stress appraisal and sleeping difficulty and negatively associated with physical performance and self‐reported cognitive resiliency in both sexes. Third, it was hypothesized that biomarker components of the ALI would help explain relationships between ALI and robust measures of physical performance and psychological responses among both sexes.

## 2 MATERIALS AND METHODS

### 2.1 Study design and participants

The United States Marine Corps (USMC) OCS is a 10‐week military physical and tactical training course for individuals seeking to become commissioned officers within the USMC. Officer candidates enrolled in this course undergo intense military and physical training during the 10‐week duration to evaluate their potential to be an officer. The current prospective cohort study represents a secondary analysis using data from a subset of men and women with archived blood and saliva samples and associated questionnaire and military physical performance data from a previously conducted study designed to identify predictors of musculoskeletal injury and resilience in USMC officer candidates (Bird et al., 2022; Koltun et al., 2022; Lovalekar et al., 2023). Data were collected during two OCS iterations from June 2022 to March 2023.

Data for the current investigation were drawn from candidates with complete biochemical, physical performance, questionnaire, and demographic data at the start and end of the training course. Participants were healthy men and women ≥18 years who provided written informed consent prior to study participation and completed the entire 10‐week training course. This study was approved by the University of Pittsburgh Institutional Review Board and Office of Naval Research (ONR) Human Research Protection Office, endorsed by the OCS Human Research Program, and performed under the Declaration of Helsinki.

### 2.2 Assessment of participant demographics, military experience, and anthropometrics

All participants were administered a self‐report questionnaire to report demographic information including sex (male or female), age (y), race/ethnicity, and whether participants enrolled in a previous OCS course and/or participated in military service prior to OCS. Participants identified their race/ethnicity as either American Indian/Alaska Native, Asian, African American, Hispanic or Latino, Native Hawaiian/Other Pacific Islander, White/Caucasian or Other. Participation in military service was assessed by participants answering a “yes” or “no” to the question “Did you have any history of prior service before entering OCS?”. Enrollment in a previous OCS course was determined by participants answering a “yes” or “no” to the question, “Is this your first enrollment in OCS?” If “no”, participants were asked to provide the number of times participants were enrolled in OCS and provide a descriptive reason. Anthropometrics were assessed using a stadiometer with a digital scale to measure height (m) and body mass (kg), respectively. Body mass index (BMI) was computed by dividing body mass by height in meters squared (kg∙(m^2^)^−1^).

### 2.3 Biological specimen collection and analysis

A total of 12 mL of whole blood was collected via standard venipuncture procedure using a 22‐ or 23‐gauge needle (Thermo Fisher Scientific Inc., Hampton, NH) and vacutainer into sterile vacuum 6 mL EDTA venous blood collection tubes and 6 mL clot‐activator venous blood collection tubes for plasma and serum, respectively. Plasma tubes were centrifuged immediately at 1500*g* for 15 min, and serum tubes were allowed to clot at room temperature for 30 min prior to centrifugation. A total of 2 mL of whole saliva was obtained via oral swab technique into cryovials (Salimetrics, LLC., Carlsbad, CA) and centrifuged at 3500*g* for 3 min. Centrifuged whole saliva, plasma, and serum were aliquoted into 1.5 mL microcentrifuge tubes (Thermo Fisher Scientific Inc., Hampton, NH) and stored on dry ice for shipment to the University of Pittsburgh Neuromuscular Research Laboratory and kept frozen at −80°C until analysis.

Serum dehydroepiandrosterone (DHEA) was analyzed via enzyme‐linked immunoassay (ELISA) (catalog # SKU: DHA31‐K01; EagleBio, Amherst, NH) with the concentration of DHEA measured using a Synergy HTX microplate reader (BioTek Instruments, Winooski, VT) with an assay sensitivity of 0.453 ng∙mL^−1^. All samples were run in duplicate with an inter‐assay and intra‐assay coefficient of variation (CV) of 8.40% and 4.13%, respectively. Serum cortisol was analyzed via ELISA (catalog #: 11‐CRLHU‐E01; ALPCO Diagnostics, Salem, NH) with cortisol concentrations assessed using a Synergy HTX microplate reader with an assay sensitivity of 0.4 μg∙dL^−1^. All samples were run in duplicate with an inter‐assay and intra‐assay CV of 3.80% and 3.76%, respectively. Serum C‐reactive protein (CRP) was analyzed via ELISA (catalog #: 30‐9710S; ALPCO Diagnostics, Salem, NH) with the concentration of CRP assessed using a light absorbance Synergy HTX microplate reader with an assay sensitivity of 1.9 ng∙mL^−1^. All samples were run in duplicate with an inter‐assay and intra‐assay CV of 8.60% and 2.70%, respectively. Salivary α‐amylase was analyzed using a chromogenic substrate, 2‐chloro‐p‐nitrophenol linked with maltotriose, with the enzymatic reaction spectrophotometrically measured via light absorbance on a microplate reader (RRID #: 5001.02 & 5001.05; Salimetrics, LLC, Carlsbad, CA) with an assay sensitivity of 0.4 U∙mL^−1^. All samples were run in duplicate with an inter‐assay and intra‐assay CV of 4.70% and 2.11%, respectively. Plasma pro‐inflammatory (interleukin‐6 [IL‐6] and tumor necrosis factor‐alpha [TNF‐α]) and anti‐inflammatory (interleukin‐10 [IL‐10]) cytokines were measured using a Simple Plex Human Multianalyte Assay kit (catalog #: ST01E‐PS‐005711; Bio‐techne, San Jose, CA) on an ELLA automated immunoassay system (Bio‐techne, San Jose, CA). Concentrations were calculated from raw values using internal standard curves created and updated by the manufacturer. The sensitivities for the cytokine assays were as follows: IL‐6: 0.28 pg∙mL^−1^, IL‐10: 0.58 pg∙mL^−1^, and TNF‐α: 0.30 pg∙mL^−1^. A volume of 25 μL of plasma was used for each assay, diluted 2× in sample buffer, and duplicates were run for each sample with an inter‐assay CV as follows: IL‐6: 3.45%; IL‐10: 3.94%; TNF‐α: 1.38%. Inflammatory cytokines recorded an intra‐assay CV ≤15% of all cytokines. Samples that were outside of the standard curve range upon first analysis were diluted and reanalyzed to verify values or assigned the assay's value for the lowest detectable limit.

### 2.4 Primary mediators of the allostatic load index

A total of eight biomarkers were used to summarize levels of physiological activity across several regulatory systems pertinent to stress‐related pathologies and quantify the ALI. The components of the ALI included measures of regulatory processes across neuroendocrine, immunological, and autonomic systems (Juster et al., 2010). These parameters included serum DHEA (a functional HPA antagonist; ng∙mL^−1^), (Juster et al., 2010; Seeman et al., 1997) serum cortisol (a functional HPA agonist; μg∙dL^−1^) (Juster et al., 2010, 2011), and serum cortisol:DHEA ratio (an index of catabolic to anabolic hormone balance; AU) (Ahmed et al., 2023); serum CRP (an immunologic pro‐inflammatory cytokine indicative of systemic inflammation; mg∙L^−1^) (Geronimus et al., 2006; Juster et al., 2010), plasma IL‐6 (immunologic pro‐inflammatory cytokine; pg∙mL) (Egorov et al., 2017; Juster et al., 2010), plasma TNF‐α (immunologic pro‐inflammatory cytokines; pg∙mL^−1^) (Bärtl et al., 2022; Hansen et al., 2021), plasma IL‐10 (immunologic anti‐inflammatory cytokine; pg∙mL^−1^) (Juster et al., 2010), and salivary α‐amylase (digestive enzyme representing sympathoadrenal activity; U∙mL^−1^) (Egorov et al., 2017; Juster et al., 2011). Currently, there is no standardized number or panel of biomarkers among specific biological systems for measuring ALI (McLoughlin et al., 2020). However, the biomarkers chosen among neuroendocrine, immunological, and autonomic systems were selected based on their employment in prior allostatic load research examining ALI in populations exposed to multi‐stressor environments, such as first responders (Giessing et al., 2020; Igboanugo et al., 2022; Kerr et al., 2021), as well as their ability to comprehensively represent biological systems most affected by military training environments (Alemany et al., 2008; Henning et al., 2014; Nindl et al., 1997; Tait et al., 2022). Change (Δ) in primary mediator values was calculated as the difference in mediator value at baseline from mediator values after training.

### 2.5 Computation of the allostatic load index

The ALI was computed using the count‐based high‐risk quartile method by leveraging the sample distribution of eight biomarkers across neuroendocrine, immunological, and autonomic systems as performed in previous studies (Carlsson et al., 2017; Rosemberg et al., 2020). This method calculates a summary measure that represents the sum of biomarkers falling beyond the 75th or below the 25th percentiles based on the sample distribution to reflect those participants displaying physiologic maladaptation to chronic stress (Rosemberg et al., 2020). High‐risk percentile values were defined as participant values greater than or equal to the 75th percentile or less than or equal to the 25th percentile based on the sample distribution (Juster et al., 2010; Seeman et al., 1997). High‐risk percentile values were calculated from the range of each biomarker, and participant biomarker values were dichotomized as 0 or 1 and aggregated into an ALI as follows: quartiles were calculated, and participant values greater than or equal to the 75th percentile were coded a score as 1, whereas values falling below the 75th percentile were coded as 0 and summed to yield an ALI. Exceptional biomarkers following this formulation included serum DHEA and plasma IL‐10, whereby values less than or equal to the 25th percentile were coded as 1, and values greater than the 25th percentile were coded as 0 (Juster et al., 2011). Biomarker component scores comprised the score (1, 0) of each biomarker that contributed to the ALI calculation. High‐risk percentile thresholds per biomarker at baseline were used for ALI calculation upon course completion as performed in previous studies (Dich et al., 2015; Juster et al., 2010).

One conceptual issue concerning the multisystem biomarkers selected in the current study that requires further investigation in military populations is the direction of maladaptation for cortisol, as both hyperactivity and hypoactivity of the HPA axis have been associated with maladaptive responses and stress‐related pathologies (Heim et al., 2000; Juster et al., 2011; Raison & Miller, 2003). Recent literature on HPA axis functioning in military physical training demonstrates inconsistent results on the direction of cortisol, with studies reporting no associations between cortisol levels and military training stress (Boesch et al., 2015) and others reporting HPA axis hyperactivity (Reini, 2010) or hypoactivity (Conkright et al., 2022; Igboanugo et al., 2022). Therefore, the cortisol and cortisol:DHEA components of the ALI were calculated, including those extreme cortisol and cortisol:DHEA values lying within either end of the at‐risk continuum (i.e., highest and lowest quartile). This method was previously conducted by Bellingrath et al. (2009) who investigated the association between ALI and exhaustion in physically and cognitively demanding occupations (Bellingrath et al., 2009).

Change in ALI scores was calculated as the difference in ALI score at baseline from participants' ALI scores upon course completion (ΔALI = ALI_post_ – ALI_pre_). Increases in ALI scores were indicative of a greater extent of allostatic load owing to physiological maladaptation and greater physiologic burden, whereas decreases in ALI scores were indicative of physiological adaptation (Rosemberg et al., 2020). Scores of ALI ranged from 0 to 8 based on the number of biomarker components used in its computation (Seeman et al., 1997). ALI scores were grouped to reflect “low” (<4) and “high” (≥4) allostatic load (Castagné et al., 2018). Change in biomarker component scores was calculated as the difference in biomarker component score at baseline from the score after training.

To date, despite evidence of sex differences in primary mediator activity in response to physical and psychological stress exposure (Juster et al., 2019; Verma et al., 2011) and during military training (Conkright et al., 2022), as well as in the downstream neurobehavioral and cardiometabolic effects evoked by HPA and SAM activation (Freire et al., 2020), there is no consensus regarding analyzing ALI or its components separately by sex (McLoughlin et al., 2020). The recent development of a gender‐integrated physical and tactical training program at OCS has served to expose both sexes to similar physical and psychological demands (Lovalekar et al., 2023) and reflects the gender‐neutral and age‐independent nature of ground‐close combat (Fitriani et al., 2016). However, notwithstanding evidence of sex differences in mediator activity in response to military training (Conkright et al., 2022), the ALI and biomarker component scores were calculated separately by sex using sex‐specific quartiles in this investigation. Criterion cutoff values for ALI computation and ranges for each of the eight biomarkers before and after training by sex are outlined in Table S1.

### 2.6 Assessment of perceived stress appraisal, cognitive resilience, and sleeping difficulty level

Participant perception of psychological stress was measured using the Perceived Stress Scale (PSS), a valid (Park & Colvin, 2019), self‐report questionnaire that measures the degree to which situations in participants' lives were perceived as stressful (Cohen et al., 1983). The PSS comprises a 10‐item scale in a Likert scale format designed to determine the extent of how unpredictable, uncontrollable, and overloaded respondents found their lives, including several direct queries about current levels of experienced stress. Each item was rated from 0 (*not at all true*) to 4 (*true nearly all of the time*). PSS global scores were obtained by reversing collected responses (i.e., 0 = 4, 1 = 3, 2 = 2, 3 = 1, and 4 = 0) to four positively stated items and then summing all scale items for a maximum possible score of 40, where higher scores indicated greater psychological stress (Cronbach's *α* = 0.59). Change in psychological stress appraisal was calculated as the difference in PSS global score at baseline from the score after training (ΔPSS = PSS_post_ – PSS_pre_).

The subjective perception of psychological resilience was measured by the Connor‐Davidson Resilience Scale (CD‐RISC‐25). The CD‐RISC‐25 is a reliable and valid (Mansfield et al., 2011) self‐rated questionnaire often administered to evaluate self‐reported resiliency and coping ability in the face of adversity. This questionnaire comprised 25 items in Likert scale format for participants to rate their responses to statements concerning how they reacted to or would have reacted to certain situations of adversity that occurred within the past month. Participants responded to statements understanding that if a particular situation had not arisen during this time, then the response would be based on how the participants thought they would have reacted or appraised themselves in reference to the particular statement. Each item was rated from 0 (*not at all true*) to 4 (*true nearly all of the time*). Global scores were calculated through the summation of all items, where the maximum possible score was 100, with higher scores indicative of greater cognitive resilience to stress (Cronbach's *α* = 0.82). Change in resilience was calculated as the difference in the global score at baseline from the score after training (ΔCD‐RISC‐25 = CD‐RISC‐25_post_ – CD‐RISC‐25_pre_).

The subjective perception of sleeping difficulty level was measured using a subset of questions from the reliable and valid Athlete Sleep Screening Questionnaire (Bender et al., 2018). Sleeping difficulty (SD) was characterized as the degree to which participants felt that they struggled to attain a sufficient night of sleep in both quality and quantity and whether they required pharmaceutical sleep aids (over‐the‐counter or prescribed) to alleviate self‐reported sleeping difficulties. Participants received a five‐item questionnaire comprising four responses to report the number of hours of actual sleep they attained in the recent past, how satisfied they were with their sleep, how long it took to fall asleep, the number of times they woke up during the night, and whether or not they took prescribed or over‐the‐counter medication for sleep. SD scores were calculated from the summation of responses where each response was graded from 0 to 4, with higher scores indicating worsened SD for a maximum score of 17 (Cronbach's *α* = 0.51). Change in SD was calculated as the difference in SD score at baseline from the score achieved after training (ΔSD = SD_post_ – SD_pre_).

### 2.7 Assessment of physical performance

Physical performance was assessed by the time to complete the three‐mile run (3MR; s), the number of pullups completed in 2 min (count), and the maximum time on a plank (s) completed during the USMC OCS Physical Fitness Test (PFT) at the start and end of the course. The PFT is a routine physical fitness test designed to assess pertinent fitness domains essential for occupational performance, including cardiorespiratory fitness and upper‐body and trunk muscular endurance. However, since January 2023, the plank exercise was authorized as the required exercise instead of crunches. Therefore, due to this change in PFT doctrine and most candidates achieving the maximum time needed for both time points (*n* = 24, 77%), as well as a few candidates performing crunches (*n* = 2, 6%) or switching exercises from pre‐ to post‐training (*n* = 3, 10%) or performing the test once (*n* = 1, 3%), neither the time to complete the plank nor the maximum number of crunches obtained were included in the current analysis. Change in the time to complete the 3MR and the number of pullups were calculated as the difference in time or count at baseline from the time or count achieved after training (Δ3MR = 3MR_post_ – 3MR_pre_; ΔPullups = Pullups_post_ – Pullups_pre_).

Additional physical performance measurements included the PFT‐derived Run‐Row score, Pushup‐Pullup score, and Crunches‐Plank score, which are alternative PFT scoring methods that comprise a standardized score based on the number of repetitions or time achieved, exercise chosen, altitude, age, and sex. All scores ranged between a minimum passing score of 40 and a maximum passing score of 100. Participants performed either pushups or pullups for the Pushup‐Pullup score, performed the 3MR or 5000‐m row for the Run‐Row score, or performed crunches or plank for the Crunches‐Plank score, wherein the number of repetitions or time achieved by age, altitude, and sex were converted to a score using the official conversion charts provided by USMC (2024). No participant in the current investigation performed the rowing or pushup components of the PFT. Therefore, the Run‐Row and Pushup‐Pullup scores were derived from 3MR and pullup performance, respectively. The total PFT score was characterized as the summation of the Pullup‐Pushup score, Run‐Row score, and Crunch‐Plank score for a minimum passing score of 120 and a maximum passing score of 300. Change in Push‐Pull score, Run‐Row score, Crunches‐Plank score, and total PFT score was calculated as the difference in scores at baseline from the scores achieved after training.

### 2.8 Confounding variables

Participant behavioral, demographic, and military service characteristics were investigated as confounders for analysis due to their influence on ALI and biomarker components observed in previous investigations. Such characteristics included smoking behavior (Juster et al., 2011), race (Deuster et al., 2011; Zare et al., 2023), and previous military service (Beckie et al., 2016; Piazza et al., 2022). Participants who had never smoked cigarettes in their lifetime were categorized as “never smokers”. Participants who had smoked cigarettes in their lifetime but do not currently smoke were categorized as “past smokers”, while participants who had smoked cigarettes in their lifetime and are currently smoking were categorized as “current smokers” (Moore et al., 2021).

### 2.9 Statistical analysis

Statistical analyses were performed using R (V.4.2.0; R Foundation for Statistical Computing, Vienna, Austria). All data were assessed for normality by the assessment of histograms and Shapiro–Wilk tests. Descriptive statistics were presented as median (Q1, Q3), count, or percent (%). Mann–Whitney *U*‐tests were conducted to determine the differences in baseline demographic and anthropometric characteristics between sexes. Wilcoxon signed‐rank tests were conducted to determine the differences in biomarkers (DHEA, cortisol, cortisol:DHEA ratio, CRP, α‐amylase, IL‐6, IL‐10, and TNF‐α), ALI, psychological measures (PSS, SD, and CD‐RISC‐25), and physical performance measures (3MR, pullups, Push‐Pull PFT score, Run‐Row PFT score, Crunches‐Plank PFT score, and total PFT score) before and after training after stratification by sex. McNemar's Test was conducted to determine the difference in the proportion of participants experiencing ‘low’ (ALI < 4) and “high” (ALI ≥ 4) allostatic load before and after training after stratification by sex. Simple linear regression analysis was conducted to determine the association between change in ALI score from baseline and change in perceived stress appraisal, resilience, sleeping difficulty, and physical performance while controlling for race, smoking behavior, and previous military service by sex. Model estimates (*β*) and coefficients of determination (*R* ^2^) with 95% confidence intervals and slope significance were reported. Backward stepwise multiple linear regression analysis was conducted to determine the association between biomarker component scores (explanatory) and physical and psychological outcomes (response) after stratification by sex. Predictors with *p*‐value <0.200 in the simple linear regression analysis were considered for inclusion in the analysis. Model estimates and coefficients of determination with 95% confidence intervals, slope significance, z‐statistic, and standard error (SE) were reported. Power analysis using G*Power (v.3.1.9.4) to determine the required sample size for multiple linear regression analysis with an *R* ^2^ value of 0.56 based on previous studies (Igboanugo et al., 2022), a significance level of 0.05, and a desired power of 0.80 indicated that approximately 15 participants per sex would be required for adequate power to detect a significant effect. Statistical significance was set a priori, at *α* = 0.05, two‐tailed.

## 3 RESULTS

### 3.1 Participants

A total of 149 participants (49 women) from two consecutive iterations from a summer (*n* = 93, 23 women) and winter OCS course (*n* = 56, 25 women) participated. After accounting for attrition (*n* = 53; 25 women), drop from OCS due to musculoskeletal injury (*n* = 17, 8 women), incomplete datasets (*n* = 73, 2 women), and biomarker concentrations with CV > 15% (*n* = 6), a total of 31 participants (14 women) were included in the current investigation. Participants were healthy young adults with a BMI classified as “pre‐obesity” for men and “normal” body mass for women based on international standards from the World Health Organization (2023). Men were taller, heavier, and had significantly higher BMIs than women (Table 1).

**TABLE 1:** Baseline characteristics of 31 marine officer candidates.

Variable	Men (*N* = 17)	Women (*N* = 14)	*p*
	Median (Q1, Q3)	Median (Q1, Q3)	
Age (y)	24.00 (21.00, 27.00)	25.50 (24.00, 27.00	0.401
Height (cm)	**176.40 (172.00, 179.20)**	**167.10 (164.20, 172.50)**	**0.001**
Body mass (kg)	**83.30 (79.80, 84.90)**	**66.60 (64.83, 69.47)**	**<0.001**
BMI (kg∙((m^−1^) ^2^)	**25.93 (25.08, 28.46)**	**23.91 (22.07, 25.25)**	**0.002**
Race/ethnicity			
American Indian/Alaska Native	0	0	–
Asian	2	1	–
African American	1	1	–
Hispanic or Latino	2	2	–
Native Hawaiian/Other Pacific Islander	0	0	–
White/Caucasian	11	9	–
Other	1	0	–
Previous military service			
Yes	6	4	–
No	11	10	–
Previous OCS enrollment			
Yes	2	4	–
No	15	10	–
Smoking behavior			
Never	13	14	–
Past	4	0	–
Current	0	0	–

*Note*: Data is shown as median (Q1, Q3) or count where appropriate; Boldface values indicate statistical significance between groups (*p* < 0.05).

### 3.2 Proportion of participants with high allostatic load at the end of OCS

Results of the proportion of participants with low (ALI < 4) and high allostatic load (ALI ≥ 4) before and after training are shown in Table 2. Six men (35.3%) had high ALI before and after training. Nine men (52.9%) began training with low ALI and ended with high ALI. One participant (5.9%) ended training with a low ALI and started with a high ALI, and one participant (5.9%) ended training with a low ALI and started with a low ALI. There was a significant increase in the proportion of men with high ALI during training (*χ* ^2^ = 4.900, df = 1, *p* = 0.027).

**TABLE 2:** Contingency table of participants with low and high allostatic load before and after military training.

After training	Before training		Total
	High allostatic load	Low allostatic load	
Males (*N* = 17)			
High allostatic load	6 (35.3%)	9 (52.9%)	15 (88.2%)
Low allostatic load	1 (5.9%)	1 (5.9%)	2 (11.8%)
Total	7 (41.2%)	10 (58.8%)	17 (100%)
Females (*N* = 14)			
High allostatic load	4 (28.6%)	4 (28.6%)	8 (57.2%)
Low allostatic load	1 (7.1%)	5 (35.7%)	6 (42.8%)
Total	5 (35.7%)	9 (64.3%)	14 (100%)

*Note*: High allostatic load is represented by an ALI value ≥4; Low allostatic load is represented by an ALI value <4. Total in the first column denotes the total number of participants who demonstrated high or low allostatic load before training with their respective percentages. Total in the final column denotes the total number of participants who demonstrated high or low allostatic load after training with their respective percentages.

For the women, a total of four women (28.6%) had high ALI before and after training. Four women (28.6%) began training with low ALI and ended with high ALI. One participant (7.1%) had a high ALI at the start of training and ended with a low ALI, whereas five women (35.7%) began training with a low ALI and ended with a low ALI (Table 2). There was no significant change in the proportion of women with high ALI during training (*χ* ^2^ = 0.800, df = 1, *p* = 0.371).

### 3.3 Comparison of ALI, biochemical, physical, and psychological outcomes before and after OCS by sex

Comparisons of ALI, biochemical, physical performance, and psychological outcomes before and after training by sex are shown in Table 3. Following the completion of OCS in men, significant increases in concentrations of serum CRP (+1.11 mg∙L^−1^, *p* = 0.003), plasma IL‐6 (+0.47 pg∙mL^−1^, *p* = 0.045) and plasma TNF‐α (+1.03 pg∙mL^−1^, *p* = 0.006), global scores on PSS (+4.00, *p* = 0.008), Run‐Row PFT score (+9.50, *p* = 0.008), and ALI (+2.00, *p* = 0.004) were observed, whereas the time to complete the 3MR significantly decreased (−81.00 s, *p* = 0.004) (Table 3). Following the completion of OCS in women, significant decreases in concentrations of serum DHEA (−1.04 ng∙mL^−1^, *p* = 0.005) and serum cortisol (pre: −2.60 μg∙dL^−1^, *p* = 0.042) and significant increases in concentrations of CRP (+1.14 mg∙L^−1^, *p* = 0.013) and plasma IL‐6 (+0.74 pg∙mL^−1^, *p* = 0.007), global score on PSS (+8.00, *p* = 0.007) and SD (+2.50, *p* = 0.007), and ALI (+1.00, *p* = 0.006) were observed (Table 3).

**TABLE 3:** Comparison of allostatic load index, biochemical, physical, and psychosocial responses before and after military training.

Variable	Males (*N* = 17)			Females (*N* = 14)		
	Before training	After training	*p*	Before training	After training	*p*
	Median (Q1, Q3)	Median (Q1, Q3)		Median (Q1, Q3)	Median (Q1, Q3)	
DHEA (ng∙mL^−1^)	3.36 (2.83, 4.15)	2.97 (2.65, 3.95)	0.548	**3.37 (2.61, 3.83)**	**2.33 (1.92, 2.60)**	**0.005**
Cortisol (ug∙dL^−1^)	7.72 (7.07, 9.25)	9.45 (6.59, 10.16)	0.431	**10.91 (8.80, 12.68)**	**8.31 (6.86, 10.51)**	**0.042**
Cortisol:DHEA	26.13 (17.03, 30.00)	25.92 (19.97, 34.21)	0.120	29.83 (22.77, 46.63)	34.56 (26.87, 42.09)	0.626
CRP (mg∙L^−1^)	**0.29 (0.13, 0.62)**	**1.40 (0.84, 4.59)**	**0.003**	**1.22 (0.53, 3.11)**	**3.97 (2.36, 5.66)**	**0.013**
AMY (U∙mL^−1^)	64.29 (35.10, 82.66)	56.42 (32.80, 92.50)	0.431	86.59 (50.92, 124.23)	85.77 (67.65, 127.02)	0.999
IL6 (pg∙mL^−1^)	**0.78 (0.63, 0.95)**	**1.25 (0.96, 1.50)**	**0.045**	**1.30 (0.96, 1.65)**	**2.04 (1.53, 3.69)**	**0.007**
IL10 (pg∙mL^−1^)	2.43 (1.80, 2.76)	2.84 (2.03, 3.37)	0.057	2.11 (1.80, 2.97)	2.25 (1.59, 3.32)	0.903
TNF‐α (pg∙mL^−1^)	**7.54 (6.76, 8.33)**	**8.57 (7.21, 9.60)**	**0.006**	6.03 (5.56, 8.28)	6.96 (5.95, 8.38)	0.576
PSS (0–40)	**10.00 (5.00, 13.00)**	**14.00 (11.00, 19.00)**	**0.008**	**11.50 (7.25, 12.00)**	**19.50 (18.00, 21.75)**	**0.007**
SD (0–17)	6.00 (4.00, 8.00)	7.00 (6.00, 7.00)	0.249	**5.50 (3.25, 6.75)**	**8.00 (8.00, 8.75)**	**0.007**
CDRISC (0–100)	80.00 (74.00, 85.00)	80.00 (72.00, 85.00)	0.887	83.50 (83.00, 88.00)	80.50 (72.25, 89.50)	0.147
3MR (s)	**1283.00 (1160.00, 1309.00)**	**1202.00 (1146.00, 1246.00)**	**0.004**	1387.00 (1367.00, 1473.00)	1407.00 (1371.00, 1434.00)	0.094
Pullups (count)	18.00 (17.00, 23.00)	19.00 (13.75, 21.50)	0.163	9.00 (8.00, 11.00)	9.00 (7.00, 11.00)	0.468
Run‐Row PFT	**78.00 (77.00, 91.00)**	**87.50 (82.25, 93.00)**	**0.008**	87.00 (78.00, 89.00)	86.00 (82.00, 93.25)	0.098
Push‐Pull PFT	90.00 (80.00, 100.00)	88.50 (69.25, 97.00)	0.330	90.00 (80.00, 100.00)	82.50 (76.25, 100.00)	0.440
Crunches‐Plank PFT	100.00 (100.00, 100.00)	100.00 (100.00, 100.00)	0.999	100.00 (100.00, 100.00)	100.00 (100.00, 100.00)	0.181
Total PFT (120–300)	275.00 (255.00, 283.00)	271.50 (257.00, 282.00)	0.483	270.00 (264.00, 280.00)	274.00 (262.20, 284.20)	0.506
ALI score (0–8)	**3.00 (2.00, 4.00)**	**5.00 (4.00, 5.00)**	**0.004**	**3.00 (2.00, 4.00)**	**4.00 (3.00, 4.75)**	**0.006**

*Note*: Values are shown as median (Q1, Q3). Boldface values indicate statistical significance (*p* < 0.05).

Abbreviations: ALI, allostatic load index; AMY, salivary alpha amylase; CDRISC, Connor‐Davidson Resilience Scale; CRP, serum c‐reactive protein; DHEA, serum dehydroepiandrosterone; IL‐10, plasma interleukin‐10; IL‐6, plasma interleukin‐6; PSS, Perceived Stress Scale; SD, sleeping difficulty subcomponent of Athlete Sleep Screening Questionnaire; TNF‐α, plasma tumor necrosis factor alpha.

### 3.4 Associations between ALI and maladaptive physical and psychological responses during training

Associations between change in ALI score and change in psychological and physical performance by sex are shown in Table 4. In men, an increased ALI score during training was associated with worsened pull‐up (*β* = −0.88, *p* = 0.015, *R* ^2^ = 0.60), Push‐Pull PFT (*β* = −2.87, *p* = 0.013, *R* ^2^ = 0.60), and total PFT performance (*β* = −3.48, *p* < 0.001, *R* ^2^ = 0.58), and a lower SD score (*β* = −0.56, *p* = 0.046, *R* ^2^ = 0.35). In women, an increased ALI score during training was associated with a lower SD score (*β* = −1.25, *p* < 0.001, *R* ^2^ = 0.82) and an improvement in CD‐RISC‐25 score (*β* = 2.65, *p* = 0.025, *R* ^2^ = 0.54) (Table 4).

**TABLE 4:** Results of simple linear regression analysis assessing the relationship between changes in allostatic load index and physical performance and psychosocial responses during military training.

Dependent variable	Males				Females			
	Independent variable: ΔALI (0–8)				Independent variable: ΔALI (0–8)			
	*β*	*R* ^2^	*p*	95% CI	*β*	*R* ^2^	*p*	95% CI
Δ3MR (s)	5.28	0.60	0.427	−8.94, 19.51	2.84	0.20	0.744	−16.53, 22.21
ΔPullups (count)	**−0.88**	**0.60**	**0.015**	**−1.55, −0.21**	−0.01	0.31	0.972	−0.71, 0.68
ΔPush‐Pull PFT Score	**−2.87**	**0.60**	**0.013**	**−4.99, −0.75**	0.20	0.19	0.903	−3.46, 3.86
ΔRun‐Row PFT Score	−0.61	0.59	0.398	−2.15, 0.93	−0.26	0.22	0.763	−2.17, 1.65
Δ Total PFT Score	**−3.48**	**0.58**	**0.007**	**−5.76, −1.19**	−1.28	0.39	0.494	−5.40, 2.84
ΔPSS (0–10)	0.04	0.52	0.960	−1.49, 1.56	−1.16	0.34	0.315	−3.62, 1.31
ΔSD (0–17)	**−0.56**	**0.35**	**0.046**	**−1.10, −0.01**	**−1.25**	**0.82**	**<0.001**	**−1.71, −0.80**
ΔCD‐RISC‐25 (0–100)	−0.71	0.37	0.401	−2.49, 1.08	**2.65**	**0.54**	**0.025**	**0.42, 4.88**

*Note*: Change in the allostatic load index (ALI) was calculated by subtracting the index value upon course completion from the index value at baseline, where a more positive change indicates an increased allostatic load from baseline. The analysis included race, smoking behavior, and previous military service as confounding variables. Boldface values indicate statistical significance (*p* < 0.05).

Abbreviations: 3MR, three‐mile run; CD‐RISC‐25, global score on Connor‐Davidson Resilience Scale; PFT, physical fitness test; PSS, Perceived Stress Scale; SD, sleep disturbance subscale from the Athlete Sleep Screening Questionnaire.

### 3.5 Associations between biomarker components and maladaptive responses during training

Results of the backward stepwise linear regression analysis evaluating ALI components associated with physical performance and psychological responses in men are shown in Table 5. An increased TNF‐α component score was significantly associated with worsened pullup (*β* = −5.52, *p* < 0.001, *R* ^2^ = 0.77) and Push‐Pull PFT performance (*β* = −15.93, *p* < 0.001, *R* ^2^ = 0.81). Additionally, an increased CRP component score (*β* = −13.95, *p* = 0.006, *R* ^2^ = 0.68) and TNF‐α component score (*β* = −11.37, *p* = 0.010, *R* ^2^ = 0.68) were significantly associated with worsened total PFT performance. Further, an increased DHEA component score (*β* = −1.56, *p* = 0.004, *R* ^2^ = 0.65) and an increased TNF‐α component score (*β* = −1.91, *p* = 0.037, *R* ^2^ = 0.65) were significantly associated with an improved SD score after training (Table 5).

**TABLE 5:** Results of backward stepwise linear regression analysis assessing the biomarker components of the ALI that are predictive of physical performance and psychosocial responses in male Marine officer candidates.

Outcome	Predictor	*β*	*R* ^2^	SE	Z	*p*‐Value	95% CI
ΔSD	Constant	3.628	0.650	0.296	1.223	1.000	−0.64, 0.64
	ΔCORT:DHEA low quartile	−1.213	0.650	0.795	−1.527	0.151	−2.93, 0.50
	**ΔDHEA low quartile**	**−1.557**	**0.650**	**0.453**	**−3.441**	**0.004**	**−2.54, −0.58**
	**ΔTNF‐α high quartile**	**−1.908**	**0.650**	**0.819**	**−2.329**	**0.036**	**−3.67, −0.14**
ΔCDRISC‐25	Constant	5.675e‐16	0.133	1.220	4.649	1.000	−2.60, 2.60
	ΔCORT:DHEA high quartile	−5.074	0.133	3.351	−1.514	0.151	−12.22, 2.07
Δ3MR	Constant	1.721	0.373	8.348	2.061	1.000	−18.03, 18.03
	ΔCRP high quartile	47.798	0.373	26.520	1.802	0.095	−9.50, 105.09
	ΔCORT low quartile	19.496	0.373	13.820	1.410	0.182	−10.36, 49.35
ΔRun‐Row PFT Score	Constant	−1.080e−16	0.411	0.880	‐1.220e‐16	1.000	−1.90, 1.90
	ΔCRP high quartile	−4.926	0.411	2.800	−1.762	0.101	−10.97, 1.12
	ΔCORT low quartile	−2.519	0.411	1.457	−1.729	0.107	−5.67, 0.63
ΔPullups	Constant	4.863e‐16	0.768	0.303	1.606	1.000	−0.65, 0.65
	**ΔTNF‐α high quartile**	**−5.521**	**0.768**	**0.810**	**−6.816**	**<0.001**	**−7.26, −3.78**
ΔPush‐Pull PFT Score	Constant	4.283e‐16	0.805	0.929	4.608	1.000	−2.01, 2.01
	**ΔTNF‐α high quartile**	**−15.931**	**0.805**	**2.600**	**−6.132**	**<0.001**	**−21.54, −10.32**
	ΔCRP high quartile	−5.950	0.805	2.913	−2.042	0.062	−12.24, 0.34
Δ Total PFT Score	Constant	1.024e‐17	0.681	1.357	7.546	1.000	−2.93, 2.93
	**ΔCRP high quartile**	**−13.948**	**0.681**	**4.254**	**−3.279**	**0.006**	**−23.14, −4.76**
	**ΔTNF‐α high quartile**	**−11.369**	**0.681**	**3.794**	**−2.997**	**0.010**	**−19.56, −3.17**

*Note*: Predictors included biomarker count‐based scoring components of the ALI (ΔDHEA low quartile, ΔCORT low quartile, ΔCORT high quartile, ΔCORT:DHEA ratio low quartile, ΔCORT:DHEA ratio high quartile, ΔCRP high quartile, Δ IL6 high quartile, ΔIL10 low quartile, ΔTNF‐α high quartile, and ΔAMY high quartile). None of the components had a *p*‐value below the threshold of 0.200 for ΔPSS during simple linear regression analysis, thereby excluding this outcome in backward stepwise regression analysis and resulting in no model output. Boldface values indicate statistical significance (*p* < 0.05).

Results of the backward stepwise linear regression analysis evaluating ALI components associated with physical performance and psychological responses in women are shown in Table 6. An increased cortisol:DHEA ratio component score (highest quartile) was associated with an improved 3MR performance (*β* = −74.53, *p* = 0.019, *R* ^2^ = 0.41), and an increased CRP component score (*β* = −8.07, *p* = 0.037, *R* ^2^ = 0.81) and IL‐6 component score (*β* = −15.80, *p* = 0.016, R^2^ = 0.81) were associated with worsened total PFT performance. Additionally, an increased IL‐10 component score was associated with an improved pull‐up (*β* = 1.50, *p* = 0.036, *R* ^2^ = 0.48) and Push‐Pull PFT (*β* = 7.64, *p* = 0.044, *R* ^2^ = 0.46) performance. Increased cortisol:DHEA ratio component score (lowest quartile) was associated with a worsened PSS score after training (*β* = 10.59, *p* = 0.006, *R* ^2^ = 0.48) (Table 6).

**TABLE 6:** Results of backward stepwise linear regression analysis assessing the biomarker components of the ALI that are predictive of physical performance and psychosocial responses in female marine officer candidates.

Outcome	Predictor	*β*	*R* ^2^	SE	Z	*p*‐Value	95% CI
ΔPSS	Constant	8.837e‐17	0.482	1.262	7.002	1.000	−2.75, 2.75
	**ΔCORT:DHEA low quartile**	**10.589**	**0.482**	**3.172**	**3.339**	**0.006**	**3.68, 17.50**
ΔSD	Constant	−2.370e‐16	0.243	0.613	−3.870e‐16	1.000	−1.34, 1.34
	ΔIL10 low quartile	−2.209	0.243	1.125	−1.963	0.073	−4.66, 0.24
ΔCDRISC‐25	Constant	5.934e‐17	0.276	1.711	3.470	1.000	−3.72, 3.72
	ΔCORT high quartile	6.941	0.276	3.247	2.137	0.054	−0.13, 14.02
Δ3MR	Constant	−2.220e‐15	0.406	9.425	−2.360e‐16	1.000	−20.75, 20.75
	**ΔCORT:DHEA high quartile**	**−74.525**	**0.406**	**27.168**	**−2.743**	**0.019**	**−134.32, −14.73**
ΔRun‐Row PFT Score	Constant	−3.820e‐16	0.508	0.885	−4.320e‐16	1.000	−1.97, 1.97
	ΔCORT:DHEA high quartile	5.884	0.508	2.778	2.118	0.060	−0.31, 12.07
	ΔDHEA low quartile	2.837	0.508	2.054	1.381	0.197	−1.74, 7.41
ΔPullups	Constant	−6.130e−17	0.478	0.330	‐1.860e‐16	1.000	−0.74, 0.74
	**ΔIL10 low quartile**	**1.502**	**0.478**	**0.621**	**2.419**	**0.036**	**0.12, 2.88**
	ΔCORT:DHEA HIGH Quartile	1.029	0.478	0.637	1.614	0.138	−0.39, 245
ΔPush‐Pull PFT Score	Constant	−4.970e‐16	0.462	1.766	−2.820e‐16	1.000	−3.93, 3.93
	**ΔIL10 low quartile**	**7.637**	**0.462**	**3.318**	**2.302**	**0.044**	**0.24, 15.03**
	ΔCORT high quartile	5.525	0.462	3.407	1.622	0.136	−2.07, 13.12
Δ Total PFT Score	Constant	2.129	0.807	1.373	1.551	1.000	−3.17, 3.17
	**ΔIL6 high quartile**	**−15.804**	**0.807**	**5.165**	**−3.060**	**0.016**	**−27.71, −3.89**
	**ΔCRP high quartile**	**−8.071**	**0.807**	**3.221**	**−2.506**	**0.037**	**−15.50, −0.64**
	ΔIL10 low quartile	4.656	0.807	3.131	1.487	0.175	−2.56, 11.88

*Note*: Predictors included biomarker count‐based scoring components of the ALI (ΔDHEA low quartile, ΔCORT low quartile, ΔCORT high quartile, ΔCORT:DHEA ratio low quartile, ΔCORT:DHEA ratio high quartile, ΔCRP high quartile, Δ IL6 high quartile, ΔIL10 low quartile, ΔTNF‐α high quartile, and ΔAMY high quartile). Boldface values indicate statistical significance (*p* < 0.05).

## 4 DISCUSSION

This investigation aimed to determine whether allostatic load quantified by the ALI significantly increased following a 10‐week military training course among male and female USMC officer candidates and determine whether the ALI was associated with maladaptive physical performance and psychological responses. This investigation also aimed to identify pertinent ALI components associated with training outcomes. Our results observed a significant increase in the proportion of men with high allostatic load following the training course (Table 2), and an increase in ALI in both sexes (Table 3), to align with our first hypothesis. However, our results observed sex‐specific associations between change in ALI and change in physical performance and psychological responses to oppose our second hypothesis (Table 4). Specifically, for the men, an increased ALI score after training was associated with a significant reduction in physical performance and self‐reported sleeping difficulty level (Table 4). In contrast, for the women, an increased ALI score after training was associated with a significant improvement in self‐reported resilience and a reduction in sleeping difficulty level (Table 4). Hence, although these results provide support for allostatic load increasing after military training to suggest a greater physiological burden experienced in both sexes (Bulmer et al., 2022), these results also suggest that allostatic load may not be associated with robust measures of maladaptation equally across sexes. Rather, the increase in allostatic load may be associated with a sex‐dependent behavioral response, rendering the ALI an indicator of worsened physical performance in men and improvement in elements of psychological well‐being in women. Further analysis revealed that reduced physical performance in men was attributed to increased TNF‐α and CRP component scores (Table 5), whereas no single or combination of component scores could explain the relationship between ALI and psychological outcomes in women (Table 6). This result did not support our third hypothesis. Collectively, these findings underscore the complexity of the allostatic load model as it may not outline an underlying biological process associated with military‐related maladaptive outcomes equally across sexes but may implicate sex‐specific behavioral responses. Military practitioners may use the ALI from this investigation to assess the physiological burden imposed on their personnel. However, given the divergent sex‐dependent behavioral relationships with ALI, robust measures of maladaptation, such as physical fitness assessments and psychological surveys, may be preferred to assess readiness for occupational role effectiveness.

### 4.1 Greater proportion of candidates with high allostatic load after training and greater impact on men

Chronic military training environments are purported to elevate allostatic load attributed to recurrent exposure to physical and psychological challenges driven by energy deficiency, sleep deprivation, cognitive distress, and strenuous physical activity, all of which may increase the presence of allostatic load wherein adaptive processes fail to react to challenges as measured by mediators of allostasis (Bulmer et al., 2022; Feigel et al., 2024). Our results observed a significant increase in the proportion of men with an ALI ≥4 at the end of training based on McNemar's test (Table 2). This suggests that military training had an overall negative impact on the number of men with high ALI as those who initially had low ALI developed higher ALI values after training. Indeed, a total of 15 men (88.2%) showed high allostatic load upon course completion. In contrast, there was no significant increase in the proportion of women with ALI values ≥4 at the end of training (Table 2). Hence, although there was an indication that women who initially had lower ALI values developed high ALI values, this change was not significant, indicating that training did not have a negative impact on allostatic load in women. Nevertheless, more than half (57.2%) of women showed high allostatic load after training. To our knowledge, this was the first study to assess changes in allostatic load status during military training and observe a greater relative impact on men than women (88.2% vs. 57.2%). This finding opposes previous studies observing higher ALI in women, even after controlling for age and race (Yang & Kozloski, 2011). However, it should be noted that the ALI should not be interpreted as a linear combination of individual systems it encompasses but rather as an indicator of biological complexity and vulnerability to stress (Fried et al., 2009). Female soldiers have been reported to be more susceptible to adverse physiological responses during military training (Conkright et al., 2022), whereas other studies report opposing views (Goldman et al., 2004). This differential may be attributed to the number and types of biomarkers assessed (Conkright et al., 2022), populations (Tait et al., 2022), and types of training conducted (O'Leary et al., 2018; Szivak et al., 2018). McLoughlin et al. (2020) reported that the ALI threshold by which allostatic load is considered remains variable and heterogeneous across studies (McLoughlin et al., 2020). Although the threshold utilized in the current investigation has been most utilized in previous investigations, there remains no standardized method to determine whether allostatic load is experienced (McLoughlin et al., 2020). Hence, a consensus regarding the influence of sex on ALI may not be possible from the current investigation. However, the ALI may overcome study discrepancies by combining stress‐related biomarkers from several systems into a single value to denote the degree of physiological maladaptation (Juster et al., 2010). Further research quantifying “high” and “low” allostatic load utilizing this threshold and their changes among personnel during training is warranted to compare results to the current investigation.

### 4.2 Allostatic load index increases following military training in both sexes

Our analysis observed a significant increase in ALI from 3.00 to 5.00 in men and 3.00 to 4.00 in women to support our first hypothesis (Table 4). Military training has been shown to alter circulating neuroendocrine, autonomic, and inflammatory biomarkers that reflect elevated or blunted primary mediator activity, which serve as key drivers of allostatic load (McEwen, 1998b). These results provide support for allostatic load increasing following military training using its traditional operationalization (Seeman et al., 1997). Previous studies reported increases in allostatic load by employing nontraditional methods for its quantification, such as nighttime heart rate variability (Corrigan et al., 2023), daytime and nighttime heart rate (Feigel et al., 2024; Logan & Barksdale, 2008), psychological stress appraisal (Bulmer et al., 2022), energy expenditure (Bobba‐Alves et al., 2022; Feigel et al., 2024), and sleep quality (Christensen et al., 2022; Feigel et al., 2024). Such measures aligned with allostatic load theory (McEwen, 1998b) but did not employ context‐relevant biomarker components for its quantification. Hence, these results may add to the literature by supporting the ALI as an additional method to quantify the cumulative physiological burden of multi‐stressor exposure (Seeman et al., 1997). Beckie et al. (2016) observed a mean ALI of 3.03 ± 2.36 comprising similar biomarker components among a large group of female veterans with multiple deployments to suggest that changes in ALI in the current investigation were comparable to individuals exposed to combat exposure (Beckie et al., 2016). Early ALI assessment during training and thereafter may serve as a beneficial health management strategy and reduce the risk of secondary and tertiary outcomes among both sexes.

### 4.3 Increased ALI is associated with sex‐specific physical performance and psychological responses

Simple linear regression analysis observed significant relationships between changes in ALI and physical performance and psychological responses unique to each sex to oppose our second hypothesis (Table 4). For the men, it was observed that increased ALI was in indirect proportion to changes in physical performance assessed by the pull‐up component of the PFT, Push‐Pull PFT score, and total PFT score (Table 4), such that greater ALI was associated with worsened physical performance outcomes. To our knowledge, this is the first investigation to determine the relationship between ALI and physical performance outcomes in a young adult population (< 40 years) or military personnel undergoing a training course, thus making comparisons between studies difficult. However, previous investigations assessing the association between ALI and physical performance outcomes assessed in mid to late‐life older adults (>48 years) report consistent results wherein higher ALI were inversely associated with various performance outcomes across balance, gait, and muscle strength domains (Germano et al., 2023; Hansen et al., 2016). Additionally, physical performance decrements during military training have been associated with altered stress hormone concentrations (Szivak et al., 2018). Szivak et al. (2018) observed a significant increase in stress hormone concentrations among personnel undergoing a military survival course and found that those who exhibited greater neuromuscular performance after training presented lower stress hormone concentrations than their unfit counterparts to suggest that fitness has a protective role on the reactivity and recovery of stress hormone concentrations (Szivak et al., 2018). Our results may reflect this phenomenon as men with higher ALI scores after training showed worsened performance outcomes (Table 5). Notably, Burley et al. (2018) observed a significant decline in physical performance among recruits who had the highest initial physical fitness levels at the start of a training course (Burley et al., 2018). However, further analysis of the men in the current investigation who had an increase or no change or decrease in ALI score after training showed similar initial fitness levels (Table S2). Hence, the relationship between increased ALI and reduced physical performance may not be influenced by fitness but attributed to changes in biomarker concentrations, including inflammatory cytokines (Table 5). Inflammatory cytokines hinder muscle protein synthesis for lean muscle mass (Miller et al., 2022) and negatively influence upper‐ and lower‐body muscular strength performance (Sharma Ghimire et al., 2023). Although the women also showed increased inflammatory cytokine concentrations (Table 3), recent evidence reports that female personnel may maintain military physical fitness due, in part, to enhanced fat oxidation during military tasks (Beckner et al., 2023). Women preferentially mobilize fat stores compared with men in response to sustained, physically demanding military training, as evidenced by increased lipid metabolites and enhanced fat oxidation, which may be beneficial for mitigating loss of lean mass (Beckner et al., 2023) and maintaining physical performance (Table 3). Further research examining the interaction of cytokine concentrations, energy utilization, and physical performance changes is warranted.

Further analysis revealed women showed a negative association between change in ALI score from baseline and change in global score on the CD‐RISC‐25, such that an increased ALI score from baseline was associated with improved self‐appraised cognitive resiliency after training (Table 4). Previous military training studies observe a beneficial response to training with improved self‐reported cognitive resiliency, as demonstrated by CD‐RISC‐25 scores in the upper quartile (McFadden et al., 2024). Additionally, military training has shown to have a positive effect on psychological resilience and reducing symptoms of depression (Guo et al., 2021). Hence, these results may support the complexity of the allostatic load model in relation to maladaptation during training by suggesting that women may respond to chronic stress differently than men, with notable benefits in the psychological domain (Conkright et al., 2022). Although not recorded in the current investigation, this phenomenon has been attributed to female personnel using coping strategies (Nakkas et al., 2016). Further research on the influence of coping strategies during training in women is warranted to confirm this hypothesis.

Interestingly, both men and women showed a negative association between change in ALI and change in self‐reported sleeping difficulty, such that increased ALI was associated with improved sleeping difficulty in both sexes (Table 4). Sleep is reported as a key driver of anabolic hormone proliferation to promote recovery, reduce inflammation from daytime stressors, and lessen the likelihood of increased allostatic load (Christensen et al., 2022). Although our findings opposed our hypothesis, this may be due, in part, to habituation of sleep during training, as observed in similar military cohorts (Larsen et al., 2022). Feigel et al. (2024) observed a median nightly sleep time (hh:mm:ss) of 05:04:59 for men and 04:53:00 for women undergoing USMC OCS without the incorporation of subjective data assessing sleep quality (Feigel et al., 2024). This sleep duration has been reported in similar USMC courses (Givens et al., 2023), and less than that observed in Australian army recruits during basic military training (Larsen et al., 2022). Hence, although this sleep duration falls well below national recommendations, the improvement in sleep difficulty may signify a habituated effect that may increase the likelihood of completing the course (Kargl et al., 2024). Further research examining sleep habituation with objective and subjective sleep data may help understand the role of sleep health and its perception on ALI in military settings (Christensen et al., 2022).

### 4.4 Outcomes associated with ALI are consistent with its components in men but not in women

Although the ALI is an established predictor of secondary and tertiary outcomes in healthy and at‐risk populations (Karlamangla et al., 2002; Logan & Barksdale, 2008), the individual biomarker components stratified by system or in combination may be differentially associated with military training‐related outcomes as well as by sex (Conkright et al., 2022). Backward stepwise regression analysis observed significant associations between biomarker components and psychological and physical performance outcomes in both sexes (Tables 5 and 6). Such components were associated with outcomes that were consistent with the ALI in men (Table 5), but not women (Table 6) to oppose our third hypothesis. For the men, an increased TNF‐α and CRP component score was associated with worsened total PFT performance after training, whereas an increase in TNF‐α component score was associated with worsened pullup and Push‐Pull PFT performance after training (Table 5). Hence, although the ALI was also able to capture these relationships (Table 4), these results support the notion that primary mediators of the immune system were among the main attributes supporting the relationship between the ALI and worsened physical performance changes in men. This result also supports the notion of breaking up the ALI into its smaller components and determining the most influential drivers related to specific outcomes (Sharma Ghimire et al., 2023). In contrast, however, our results observed opposing results in the women wherein individual or combinations of biomarker components were not consistent with ALI outcomes (Table 6). Hence, although the ALI was able to indicate adaptive psychological benefits in women (Table 4), individual components of the ALI may not relate to the same robust outcomes as observed with the ALI. This differential may be attributed to the ALI serving as a single index of eight scores representing at‐risk primary mediator values in which may vary in composition by physiological system by sex during military training (Conkright et al., 2022). Further investigation assessing the role of components of the ALI on physical performance and psychological outcomes by sex using larger sample sizes is warranted to support these findings.

### 4.5 Strengths and limitations

Although this investigation unveiled novel results, it is not without limitations. First, it is important to recognize that there is significant heterogeneity in the operationalization of ALI, which may hinder its comparison to previous studies (Parker et al., 2022). To mitigate this limitation, the current investigation computed the ALI by leveraging the most common biomarkers used in its computation, as identified in a recent literature review (Juster et al., 2010) and employing stress‐related biomarkers responsive to military training environments (Henning et al., 2014; Kargl et al., 2024; Tait et al., 2022). However, recent work suggests that associations with ALI may be robust to differences in its score composition to suggest that some findings may be replicated (Kezios et al., 2022). Nevertheless, there is a need for future work to establish a consensus operationalization of ALI specific to populations and outcomes (McLoughlin et al., 2020).

Second, since the development of the count‐based high‐risk quartile method to compute the ALI (Seeman et al., 1997), there have been numerous algorithms involving different biomarkers, biological systems, and threshold values based on clinical reference ranges or sample distributions (Juster et al., 2010). The inception of the ALI based on clinical reference ranges from general population normative values unveiled the limitation in applying this approach for the current investigation comprising a sample of military personnel (Juster et al., 2010). Military personnel face distinct and unique stressors compared to the general population that may elicit unique physiological and psychological responses. Consequently, the use of clinical‐based thresholds for ALI computation may have inadequately captured these dynamics and led to inaccuracies in assessing allostatic load. The count‐based high‐risk quartile method was used to overcome these limitations by calculating ALI from biomarkers based on a specific population (Karlamangla et al., 2002; Seeman et al., 1997). This approach was similarly performed in previous investigations examining changes in ALI under occupational stressors (Carlsson et al., 2017). Hence, by aligning the method of ALI computation with the stress profiles of the current sample, the count‐based high‐risk quartile method was the chosen method to provide greater relevance and specificity for a military sample (Beckie et al., 2016; Piazza et al., 2022; Trousselard et al., 2021).

Third, the current investigation was conducted on a relatively small sample size of 31 Marine officer candidates, which, although consisting of approximately 50% women, may have underpowered the results based on the power analysis. Indeed, a total of 47% of the original sample was found to attrit from the training course and 11.4% failed to complete the course owing to musculoskeletal injury, which may be attributed to USMC OCS being among the highest attritting and physically demanding USMC courses (Forse et al., 2024). Nevertheless, this sample provided a comprehensive dataset across the psychological, physical performance, and biochemical domains and completed the course to calculate a clinical index score in a military training setting for the first time. Further investigation assessing the ALI during military training and examining its relationship with secondary outcomes with larger samples of both sexes is warranted.

Fourth, the results may reveal shortcomings of the ALI as a singular clinical index of allostatic load that increases the risk of secondary outcomes due to the responses observed in the women (Table 4). As a singular index, the ALI is thought to be useful for military training due to the multiple stressors encountered in which may reveal a single value for ease of interpretation reflecting physiological burden (Table 3). However, the association between secondary outcomes and ALI may not be linear, as supported by individual biomarker components contributing to by‐system outcomes compared to ALI, as shown in the women (Tables 5 and 6). Women showed significant increases in HPA and immune activity after training, which may have driven ALI (Table 3). However, other studies show adverse cardiovascular, metabolic, and anthropometric responses to training in women compared to men (Gifford et al., 2021; Taylor et al., 2014). Hence, although the ALI comprised the main primary mediators (McEwen, 1998b), exploration of mediators from cardiovascular (i.e., systolic blood pressure), metabolic (i.e., low‐density lipoprotein), and anthropometric (i.e., waist‐hip ratio) domains is warranted. Further, assessing post‐training readiness through a comprehensive evaluation of multiple biomarker domains may require a multivariate approach that integrates multiple physiological inputs with variable, potentially sex‐specific, thresholds. Koltun et al. highlighted the value of moving beyond singular biomarker predictors to leveraging multiple biomarkers to better understand key outcomes. Applying supervised or unsupervised machine learning techniques with large sample sizes could enhance the utility of biomarker profiles to offer a more robust approach for predicting outcomes during military training (Koltun et al., 2023).

Fifth, the authorized change in the OCS PFT components during this investigation, particularly the transition from employing the crunches exercise to a time‐to‐complete plank exercise, as well as the difference in the number of participants who performed only crunches or switched between exercises, caused these individual components to be removed from the analysis. However, our analysis retained the PFT‐derived Crunches‐Plank score as a standard measure based on the performance of crunches or plank exercises to allow a comprehensive evaluation of physical fitness assessed during military training.

## 5 CONCLUSION

Quantifying allostatic load using its traditional operationalization on military personnel undergoing military training demonstrates a significant increase in personnel upon course completion to indicate a greater physiological burden and risk of secondary outcomes characterized by physical and psychological maladaptation in both sexes. However, the allostatic load model may not outline a biological process driving robust secondary outcomes in both sexes. Rather, increased ALI may be associated with worsened physical performance changes in men, attributed to increased biomarker component scores of the immune system, whereas increased ALI in women may be associated with improved psychological outcomes, with no specific component or combination thereof explaining this relationship. Hence, these results may suggest that increased allostatic load from chronic military training stress may accompany a potential sex‐dependent behavioral response that increases the risk for physical performance decrements in men and opportunity for improving psychological outlook in women. Military practitioners may use the sex‐specific ALI methodology from this investigation to assess the physiological burden imposed on their personnel. However, given the potential sex‐dependent behavioral relation with ALI, robust measures of maladaptation, such as routine physical fitness and psychological assessments, may be preferred to monitor physical and psychological adaptation and support sufficient post‐training occupational role performance.

## AUTHOR CONTRIBUTIONS

EF was involved in conceptualization, data curation, formal analysis, writing—original draft preparation, and visualization. KK was involved in conceptualization, methodology, investigation, resources, writing—review and editing, supervision, and project administration. ML was involved in methodology, formal analysis, and writing—review and editing. CK was involved in data curation and writing—review and editing. MB was involved in conceptualization, methodology, investigation, resources, data curation, writing—review and editing, supervision, and project administration. JF was involved in data curation, methodology, and writing—review and editing. VP was involved in data curation and methodology. BM was involved in methodology, investigation, resources, funding acquisition, supervision, and project administration. EN and KF were involved in writing—review and editing. BN was involved in conceptualization, methodology, resources, writing—review and editing, supervision, project administration, and funding acquisition.

## FUNDING INFORMATION

The sources of outside support that were acquired toward the completion of this research project include ONR N00014‐21‐1‐2725, and the views presented from this work are those of the authors and do not necessarily represent the views of the Department of Defense or its components.

## CONFLICT OF INTEREST STATEMENT

Authors disclose no professional relations among the companies or manufacturers involved in the data collection of the current study, and the results of the current study do not constitute endorsement by ACSM. Authors declare that the results of the current study were presented clearly, honestly, and without fabrication, falsification, or inappropriate data manipulation.

## ETHICS STATEMENT

All study procedures were approved by the University of Pittsburgh Institutional Review Board and Office of Naval Research (ONR) Human Research Protection Office, endorsed by the OCS Human Research Program, and performed under the Declaration of Helsinki. Written and verbal consents were obtained from each participant before they partici‐pated in this study.

## Supporting information


Table S1.

Table S2.

## Data Availability

The data that support the findings of this study are available upon reasonable request from the study principal investigator (BN), but restrictions apply to the availability of these data, which were used under license for the current study and so are not publicly available.
