# Supplementary material for: Association of allostatic load measured by allostatic load index on physical performance and psychological responses during arduous military training
Source: Physiol Rep. 2025 Mar 20;13(6):e70273. doi: 10.14814/phy2.70273 (PMC11923871; doi:10.14814/phy2.70273)
Supplement: Supplementary file 1 — Table S1. [file PHY2-13-e70273-s002.docx]

| Supplement 1. Criterion cutoff values of the eight biomarkers of the allostatic load index amongst 31 Marine Officer Candidates. | | | | | | | |
| --- | --- | --- | --- | --- | --- | --- | --- |
|  | Baseline Values | | | | |  |  |
|  | Min | 25^th^ Percentile | 50^th^ Percentile | 75^th^ Percentile | Max | Proportion Meeting or Beyond Cutoff at Baseline* | Proportion Meeting or Beyond Cutoff after Training* |
| Males (N = 17) |  |  |  |  |  |  |  |
| Biomarker (unit) |  |  |  |  |  |  |  |
| Serum DHEA (ng∙mL^-1^) | 2.047 | 2.831* | 3.360 | 4.153 | 7.657 | 4 (23.5%) | 1 (5.9%) |
| Serum Cortisol (ug∙dL∙^-1^) | 5.822 | 7.074* | 7.719 | 9.253* | 14.643 | 10 (58.8%) | 9 (52.9%) |
| Serum Cortisol:DHEA Ratio | 10.65 | 17.03* | 26.13 | 30.00* | 37.71 | 10 (58.8%) | 8 (47.1%) |
| Serum CRP (mg∙L^-1^) | 0.051 | 0.135 | 0.291 | 0.625* | 9.158 | 2 (11.8%) | 4 (23.5%) |
| Salivary α-Amylase (U∙mL^-1^) | 1.968 | 35.096 | 64.288 | 82.656* | 172.856 | 3 (17.6%) | 3 (17.6%) |
| IL-6 (pg∙mL^-1^) | 0.560 | 0.630 | 0.780 | 0.950* | 3.160 | 3 (17.6%) | 2 (11.8%) |
| IL-10 (pg∙mL^-1^) | 1.160 | 1.800* | 2.430 | 2.760 | 3.780 | 4 (23.5%) | 3 (17.6%) |
| TNF-α (pg∙mL^-1^) | 3.940 | 6.760 | 7.540 | 8.330* | 9.290 | 4 (23.5%) | 6 (35.3%) |
| Females (N = 14) |  |  |  |  |  |  |  |
| Biomarker (unit) |  |  |  |  |  |  |  |
| Serum DHEA (ng∙mL^-1^) | 1.567 | 2.605* | 3.369 | 3.825 | 7.317 | 4 (28.6%) | 10 (71.4%) |
| Serum Cortisol (ug∙dL∙^-1^) | 5.417 | 8.800* | 10.912 | 12.678* | 33.741 | 8 (57.1%) | 10 (71.4%) |
| Serum Cortisol:DHEA Ratio | 19.08 | 22.77* | 29.83 | 46.63* | 93.88 | 8 (57.1%) | 5 (35.7%) |
| Serum CRP (mg∙L^-1^) | 0.217 | 0.531 | 1.217 | 3.109* | 13.836 | 4 (28.6%) | 8 (57.1%) |
| Salivary α-Amylase (U∙mL^-1^) | 35.42 | 50.92 | 86.59 | 124.23* | 213.86 | 4 (28.6%) | 4 (28.6%) |
| IL-6 (pg∙mL^-1^) | 0.560 | 0.958 | 1.295 | 1.648* | 2.640 | 4 (28.6%) | 9 (64.3%) |
| IL-10 (pg∙mL^-1^) | 1.160 | 1.800* | 2.110 | 2.965 | 5.810 | 4 (28.6%) | 5 (35.7%) |
| TNF-α (pg∙mL^-1^) | 4.740 | 5.555 | 6.030 | 8.280* | 9.710 | 4 (28.6%) | 4 (28.6%) |
| *Note*. The allostatic load index (ALI) was calculated by summing coded biomarkers falling within high-risk quartiles based on the sample distribution. Asterisks indicate the high-risk quartile thresholds where values above the 75^th^ percentile or below the 25^th^ percentile would be counted and summed for the index value. The high-risk frequency column denotes the number of participants within the at-risk quartile. Min = minimum biomarker value; 25% = 25^th^ percentile; 50% = median; 75% = 75^th^ percentile; Max = maximum biomarker value. | | | | | | | |
