# Supplementary material for: Association of allostatic load measured by allostatic load index on physical performance and psychological responses during arduous military training
Source: Physiol Rep. 2025 Mar 20;13(6):e70273. doi: 10.14814/phy2.70273 (PMC11923871; doi:10.14814/phy2.70273)
Supplement: Supplementary file 2 — Table S2. [file PHY2-13-e70273-s001.docx]

| Supplement 2. Comparison of initial physical performances between males who showed no change or decrease (↔↓) in the allostatic load index (ALI) and males who showed an increase (↑) in ALI. | | | | |
| --- | --- | --- | --- | --- |
| Variable | ↔↓ ALI (N = 12) | ↑ALI (N = 5) | U | *p*-value |
| 3MR | 1289.00 (1203.00, 1311.00) | 1281.00 (1140.00, 1308.00) | 25.00 | 0.635 |
| Push-Pull PFT score | 83.00 (79.25, 100.00) | 100.00 (90.00, 100.00) | 41.00 | 0.250 |
| Crunches-Plank PFT score | 100.00 (100.00, 100.00) | 100.00 (100.00, 100.00) | 30.00 | 0.998 |
| Total PFT score | 264.50 (250.50, 279.20) | 277.00 (275.00, 287.00) | 42.50 | 0.205 |
| Pullups | 18.00 (16.75, 23.00) | 23.00 (20.00, 23.00) | 39.00 | 0.362 |
| Run-Row PFT score | 78.00 (75.75, 87.25) | 79.00 (77.00, 94.00) | 36.00 | 0.561 |
| *Note*. Median (Q1, Q3) values are shown between groups. | | | | |
